# Effects of cognitively engaging physical activity interventions on executive function in children and adolescents: a systematic review and meta-analysis

**DOI:** 10.3389/fpsyg.2024.1454447

**Published:** 2024-08-23

**Authors:** Fan Mao, Fang Huang, Shan Zhao, Qun Fang

**Affiliations:** ^1^School of Physical Education, Qingdao University, Qingdao, China; ^2^Research Center for Youth Football, Qingdao University, Qingdao, China; ^3^Department of Neurology, Louisiana State University Health Shreveport, Shreveport, LA, United States

**Keywords:** physical activity, cognitive challenge, cognitive function, children, adolescent

## Abstract

**Background:**

This systematic review and meta-analysis aim to evaluate the effects of cognitively engaging Physical Activity (PA) interventions on Executive Function (EF) in children and adolescents. It examines how different intervention modalities, durations, frequencies, and session lengths influence these effects.

**Methods:**

We followed the PRISMA guidelines and searched PubMed, SPORTDiscus, Embase, and Web of Science for relevant studies. Studies were included if they were Randomized Controlled Trials (RCTs) focusing on PA with cognitive elements targeting EF in healthy children and adolescents. Data were extracted and effect sizes computed using Standardized Mean Differences (SMDs).

**Results:**

From an initial 1,635 articles, 23 studies with 2,857 participants were included. The overall effect of cognitively engaging PA on EF was significant (SMD = 0.32, 95% CI 0.14–0.51), with notable improvements in inhibitory control (SMD = 0.35) and working memory (SMD = 0.34). High heterogeneity was observed (*I*^2^ = 91.1%). Moderator analyses revealed that interventions lasting more than 6 weeks, with sessions over 20 min and conducted more than twice a week, were particularly effective.

**Conclusion:**

Cognitively engaging PA interventions positively impact EF in children and adolescents, particularly in inhibitory control. Effective interventions are characterized by longer duration, higher frequency, and extended session lengths. These findings underscore the importance of integrating cognitive challenges within PA programs to enhance EF, warranting future research and practical applications in educational and developmental settings.

## 1 Introduction

Executive Function (EF) refers to a series of interrelated higher-order cognitive processes responsible for cognitive regulation and the adaptive control of goal-directed behavior (Diamond, 2013). This cognitive process is particularly triggered in situations that require concentrated attention, present challenges, and involve complexity (Diamond and Ling, 2016). The utilization of EFs prompts individuals to shift cognitive strategies, inhibit impulsive responses, and engage in planning and action (Diamond, 2012; Diamond and Ling, 2020). Core EFs comprise inhibitory control, cognitive flexibility, and working memory (Diamond, 2015; Miyake et al., 2000), which has been proved a predictor of school readiness and academic achievement for children and adolescents (Blair and Diamond, 2008; Roebers et al., 2014). Furthermore, related studies have found EF to be negatively correlated with a broad range of school-related behavioral issues (Espy et al., 2011; Friedman et al., 2007). Espy et al. (2011) established the Problem Behavior-Executive Control Model in which executive control is negatively correlated to hyperactive behaviors (*r* = −0.49, *p* < 0.05), attention problems (*r* = −0.55, *p* < 0.05), and disinhibition behaviors (*r* = −0.48, *p* < 0.05). Friedman et al. (2007) examined correlations between behavioral problems and EFs across ages. The patterns of correlations remained consistent over years, with statistically significant correlations between attention problems and EFs including inhibition (*r* = −0.41), updating (*r* = −0.24), and shifting (*r* = −0.15). Given the correlations between EF and educational outcomes as well as daily behavior, the question about how to enhance cognitive performance in children and adolescents becomes increasingly important.

EF development involves a complex interplay of genetic, environmental, and experiential factors (Diamond, 2013). One such factor that has gained an increasing attention in recent years is Physical Activity (PA) and its potential to enhance EF (Hillman et al., 2008). PA interventions have been proposed as a promising avenue to EF promotion for children and adolescents due to the overlapping neural networks and physiological mechanisms involved in both PA and EF (Best, 2010). A meta-analysis investigated the effects of different types of PA on cognitive function. The synthesized findings indicated that aerobic exercise, motor skill training, and cognitively engaging PA have positive effects on cognitive performance (Vazou et al., 2019). However, not all forms of PA interventions induce comparable improvement in cognitive performance. According to the research by Diamond and Ling (2020), interventions that focus solely on increasing energy expenditure without considering cognitive demands have relatively modest effects on EF. Therefore, interventions involving PA should adopt a comprehensive approach to student development, targeting multiple objectives such as physical health and psychological well-being (Best, 2010; Tomporowski et al., 2011).

Empirical evidence has shown that cognitively engaging PA interventions, which combine physical exertion with cognitive challenges, may be particularly effective in enhancing EF in youth (Best et al., 2011). Cognitively engaging PA interventions involve activities that require both physical effort and cognitive engagement, such as aerobic exercise combined with tasks demanding attention, memory, or problem-solving (Hillman et al., 2009; Schmidt et al., 2020). This approach highlights a shift from “simple movement to thinking moves” (Diamond, 2015). According to this view, PA programs not only focus on health-related outcomes and energy expenditure, but also consider promoting both physical and cognitive development (Pesce, 2012; Pesce et al., 2018).

Applications of neuroimaging technique facilitated investigating exercise-induced cognitive promotion at the neural level. A recent study via functional near-infrared spectroscopy identified enhanced inhibitory control after acute physical activity (Ludyga et al., 2024). Specifically, faster reaction and higher accuracy in the Stroop task were associated with decreased lateral oxygenation difference in the cognitive trials, suggesting an increased neural efficiency in the left prefrontal cortex of the participants (*N* = 29, age = 11.1 years). Another study employing electroencephalography examined brain activation changes after a 20-week exercise program for children with overweight and obesity (Mora-Gonzalez et al., 2024). Significant changes were identified when performing the assigned cognitive tasks, suggesting exercise-induced modulations in brain activity which facilitated working memory and inhibitory control of the children.

Despite increasing body of literature investigating the effects of cognitively engaging PA interventions on EF in children and adolescents, the findings of the existing studies are inconsistent. Some studies have reported significant improvements in EF following participation in cognitively engaging PA interventions (Benzing et al., 2016; Egger et al., 2019; Schmidt et al., 2015), while others reported non-significant results (Bedard et al., 2021; Mavilidi et al., 2023; Meijer et al., 2020), with some even reporting negative effects (Egger et al., 2018; van der Fels et al., 2020). These discrepancies may be attributed to methodological variations across studies, including differences in intervention protocols, duration, frequency of exercise, outcome measures, and participant characteristics. A systematic review analyzed the effects of cognitive engaging PA on EF in children, incorporating findings from 11 articles. The results indicated a moderate effect size of cognitive engaging PA on EF, particularly in the domains of working memory and cognitive flexibility (Song et al., 2022). However, the limited number of studies included in this review may lead to potential misinterpretation of the results. Additionally, the lack of research focusing on adolescent populations suggests potential limitations in the generalizability of the findings. Moreover, the underlying mechanisms through which cognitively engaging PA influences EF are still not fully understood, warranting further investigation.

The aim of this paper is to provide a comprehensive meta-analysis regarding the specific effects of cognitively engaging PA interventions on EF in children and adolescents. Moderator analyses are conducted to understand the potential moderating effects of intervention modalities, duration, exercise frequency, and outcome measures on the EFs. By synthesizing data from diverse studies and quantitatively analyzing effect sizes, a systematic review and meta-analysis can offer valuable insights into the magnitude and consistency of the effects of cognitively engaging PA interventions on EF, thus informing future research directions and practical applications in educational settings.

## 2 Methods

This meta-analysis was performed following the guidelines of Preferred Reporting Items for Systematic Reviews and Meta-Analyses (PRISMA) statement (Moher et al., 2015) and Cochrane Collaboration handbook (Cumpston et al., 2019).

### 2.1 Search strategy

The electronic databases PubMed, SPORTDiscus, Embase, and Web of Science were searched for original research published in peer-reviewed journals by March 2024. According to the main purpose of the current review, which aims to examine influence of cognitively engaging PA intervention on cognitive performance of children and adolescents, the keywords for literature search involve four categories including cognitive, physical activity, executive function, and population. Specifically, combinations of the four categories of MeSH terms were “cognitively engaging OR cognitive engagement OR cognitive demand OR cognitively challenging OR cognitive challenge” AND “physical activity OR exercise OR exergaming” AND “executive function OR inhibition OR working memory OR updating OR cognitive flexibility OR shifting” AND “adolescent OR child OR teen OR youth.”

### 2.2 Eligibility criteria

Inclusion criteria included publication in peer review journals in the English language. The language was restricted to English. Further requirements on the eligible criteria followed the guide of Population, Intervention, Comparison, Outcomes, and Study (PICOS). First, participants of the included studies should be healthy children and adolescents. Second, intervention was designed PA with cognitive elements. Third, comparisons were made between intervention and control groups at different time points. Fourth, outcomes measured EF such as cognitive flexibility, working memory, inhibitory control, etc. Fifth, the included studies must be Randomized Control Trials (RCTs).

Studies were excluded for any of the following reasons: (1) review, book chapter, commentaries, or proceedings; (2) articles in which data could not be obtained or extracted for estimating effect size even after contacting the authors; (3) non-target population; (4) studies only included an experimental group, lacking both intervention and control group comparisons.

The initial search was screened by an overall examination on the title to remove duplicates and irrelevant articles. The second phase of screening was conducted by analysis on the abstracts. For the articles that passed the first two phases, a full-text evaluation was performed to determine the included studies of the current review. Two authors (FM and QF) independently worked on the literature screening and selection. Any disagreement on the eligibility of an article was resolved by discussing with other authors to reach a consensus.

### 2.3 Data extraction and synthesis

The data of publication year, participant description, study design, instrument, EF variables, settings and interventions of eligible studies were extracted and summarized in Table 1.

**Table 1:** Characteristics of the included studies.

Study	Participant description		Design	Instrument	EF variables	Settings	Interventions	
	Mean age (years)	*N*					Intervention arm	Control arm
Benzing et al. (2016)	EG: 14.5 CG: 14.4	EG: 21 CG: 21	RCT	DF	Working memory; cognitive flexibility; inhibitory control	Exergaming	Cognitive engagement exergaming; 15 min	None
Bulten et al. (2022)	EG: 12.1 CG: 11.7	EG: 13 CG: 13	RCT	Stroop task; TMT; FWMT	Working memory; cognitive flexibility; inhibitory control	PA games/sports	Dual task PA games; 20 min	Running; 20 min
Chou et al. (2020)	EG: 12.3 CG: 12.1	EG: 44 CG: 40	RCT	Stroop task	Inhibitory control	PA games	Cognitive PE lessons; 40 min/session, 3 times/wk., 8 weeks	Original PE lessons; 40 min/session, 3 times/wk., 8 weeks
Crova et al. (2014)	EG: 9.6 CG: 9.6	EG: 37 CG: 33	RCT (cluster)	RNG	Working memory; inhibitory control	FMS	Enhanced PE program; 120 min/session, 1 time/wk., 21 weeks	Regular PE lessons; 120 min/session, 1 time/wk., 21 weeks
Egger et al. (2018)	EG: 8.0 CG: 7.9	EG: 59 CG: 54	RCT (cluster)	Flanker task; BCR	Working memory; cognitive flexibility; inhibitory control	FMS	Cognitive FMS; 20 min	None
Egger et al. (2019)	EG: 7.9 CG: 8.0	EG: 47 CG: 49	RCT (cluster)	Flanker task; BCR	Working memory; cognitive flexibility; inhibitory control	FMS	Cognitive PA; 10 min/session, 2 times/wk., 20 weeks	Aerobic PA; 10 min/session, 2 times/wk., 20 weeks
Flynn and Richert (2017)	EG: 9.2 CG: 8.8	EG: 35 CG: 41	RCT	Flanker task;	Working memory; cognitive flexibility; inhibitory control	Exergaming	Cognitive engagement exergaming; 20 min	None
Gao et al. (2019)	EG: 4.6 CG: 4.9	EG: 18 CG: 14	RCT	DCCS	Cognitive flexibility	Exergaming	Cognitive engagement exergaming; 30 min/session, 5 times/wk., 12 weeks	Regular PA; 12 weeks
Giordano and Alesi (2022)	5.7	EG: 25 CG: 25	RCT (cluster)	Stroop task	Inhibitory control	FMS	Cognitive engaging PA; 15 min/session, 3 times/wk., 6 weeks	None
Jäger et al. (2015)	EG: 11.2 CG: 11.3	EG: 54 CG: 58	RCT	Flanker task; n-back	Working memory; cognitive flexibility; inhibitory control	PA games	Cognitive engagement PA; 20 min	None
Kolovelonis and Goudas (2023)	10.1	EG: 36 CG: 32	RCT (cluster)	DF	Working memory; cognitive flexibility; inhibitory control	FMS	Cognitive challenging PA; 45 min	Waitlist
Layne et al. (2020)	8–9	EG: 19 CG: 21	RCT (cluster)	Go/NoGo task	Inhibitory control	Exergaming	Cognitively engagement exergaming; 10 min/session, 5 times/wk., 4 weeks	Waitlist
Mavilidi et al. (2023)	EG: 4.3 CG: 4.4	EG: 54 CG: 39	RCT (cluster)	Go/NoGo task; Card Sort; Mr. Ant	Working memory; cognitive flexibility; inhibitory control	FMS	Cognitively engaging PA; 17 min/session, 2 times/wk., 6 weeks	None
Meijer et al. (2020)	EG: 9.0 CG: 9.2	EG: 235 CG: 415	RCT (cluster)	DST; SST	Working memory; inhibitory control	PA games	Cognitively demanding exercise; 30 min/session, 4 times/wk., 14 weeks	Regular PE lessons; 30–45 min, 2 times/weeks
Meijer et al. (2022)	EG: 9.1 CG: 9.2	EG: 32 CG: 31	RCT (cluster)	DST	Working memory	PA games	Cognitively demanding exercise; 30 min/session, 4 times/wk., 14 weeks	Regular PE lessons; 30–45 min, 2 times/weeks
Oppici et al. (2020)	EG: 8.8 CG: 8.9	EG: 30 CG: 20	RCT	Flanker task; DCCS; LSWM	Working memory; cognitive flexibility; inhibitory control	FMS	Cognitively engaging PE; 60 min/session, 2 times/wk., 7 weeks	Regular PE lessons; 2 times/wk
Pesce et al. (2016)	5–10	EG: 232 CG: 228	RCT (cluster)	RNG	Working memory; inhibitory control	FMS	Cognitively engaging PE; 60 min/session, 1 time/wk., 24 weeks	Regular PE lessons; 60 min/session, 1 time/wk., 24 weeks
Robinson et al. (2022)	EG: 15.8 CG: 15.8	EG: 24 CG: 23	RCT (cluster)	Flanker task; DCCS; PSMT	Working memory; cognitive flexibility; inhibitory control	FMS	Cognitively demanding exercise; 8 min/session, 3 times/wk., 4 weeks	None
Schmidt et al. (2015)	EG: 11.3 CG: 11.4	EG: 69 CG: 55	RCT (cluster)	Flanker task; n-back	Working memory; cognitive flexibility; inhibitory control	PA games	Cognitive PE program; 45 min/session, 2 times/wk., 6 weeks	Regular PE lessons; 45 min/session, 2 times/wk., 6 weeks
Schmidt et al. (2020)	EG: 5.4 CG: 5.3	EG: 75 CG: 62	RCT (cluster)	n-back; DCCS SLDN	Working memory; cognitive flexibility; inhibitory control	PA games	Cognitively engaging PA; 15 min/session, 4 times/wk., 6 weeks	Waitlist
van der Niet et al. (2016)	EG: 8.8 CG: 8.9	EG: 47 CG: 52	RCT	Stroop task; VMS; TMT	Working memory; cognitive flexibility; inhibitory control	PA games	Cognitively engaging PA; 30 min/session, 2 times/wk., 22 weeks	None
van der Fels et al. (2020)	EG: 8.8 CG: 8.8	EG: 19 CG: 47	RCT (cluster)	Stop-signal	Inhibitory control	PA games	Cognitively engaging PA; 35 min	Regular PE lessons; 35 min
Vazou and Mavilidi (2021)	EG: 4.3 CG: 4.1	EG: 130 CG: 129	RCT	SLDN	Inhibitory control	FMS	Cognitively engaging PA; 10 min/session, 5 times/wk., 8 weeks	Regular PE lessons; 10 min/session, 5 times/wk., 8 weeks

CG, control group; EG, experimental group; DF, design fluency; TMT, trail making test; FWMT, forward working memory task; RNG, random number generation; BCR, backward color recall task; DST, digit span task; SST, stop signal task; DCCS, dimensional change card sort; LSWM, list sorting working memory; PSMT, picture sequence memory test; SLDN, Stroop like day night; VMS, visual memory span test; FMS, fundamental movement skill; PA, physical activity; PE, physical education; EF, executive function; RCT, randomized controlled Trial; N, number of participants.

The quantitative data were synthesized according to the categories of cognitive functions. Effect sizes which assessed the same cognitive domain were combined to compute an overall effect. Multiple effects in the same study were addressed by the following steps. First, if multiple results were reported by one cognitive assessment, the result of the more cognitive demanding condition was extracted (Álvarez-Bueno et al., 2017; Xue et al., 2019). Second, for the tests which reported both accuracy and time, accuracy was selected as a representation of cognitive performance. Therefore, in the study which applied Flanker task to assess inhibitory control, accuracy of incongruent trials would be extracted in data synthesis.

### 2.4 Statistical analysis

Statistical analysis was conducted in Comprehensive Meta-Analysis 3.3 (BioStat Inc., Englewood, NJ, United States). Considering the different outcomes and units of cognitive measures used in the studies, Standardized Mean Differences (SMD) of pre-post intervention were calculated and given weight by its inverse variance. The magnitude of the effect sizes was assessed using Cohen’s *d* values, with SMD values of <0.2, 0.2 ≤ SMD < 0.5, and 0.5 ≤ SMD < 0.8 indicating small, moderate, and large effect sizes, respectively (Cumpston et al., 2019). Quantitative pooled analysis based on a fixed-effects model, and a random-effects model if heterogeneity exists. To assess the degree of statistical heterogeneity in the meta-analysis, we employed the *I*^2^ statistic and *p*-value. Specifically, *I*^2^ values of 25, 50, and 75% were used to indicate small, moderate, and large levels of heterogeneity, respectively (Egger et al., 1997). Egger’s regression test was performed to assess publication bias in the reviewed literature. A two-tailed test with p-value less than 0.05 was considered significant publication bias.

Subgroup analyses based on three core EFs domains (cognitive flexibility, inhibitory control and working memory) were conducted after the overall meta-analysis. Intervention modality (Fundamental movement skills-FMS, PA games and exergaming), duration (>6 weeks vs. ≤6 weeks), session length (>20 min vs. ≤20 min), and frequency of intervention (>2 times/week. vs. ≤2 times/week) were examined by subgroup analyses and moderator analysis.

### 2.5 Risk of bias assessment

Two authors (FM and QF) independently assessed the risk of bias of included studies following the PEDro scale (Maher et al., 2008). The summary of the quality assessment was presented in Table 2. Any disagreements were discussed with a third author (SZ) until a consensus was achieved.

**Table 2:** Quality assessment of included studies.

Study	Eligibility criteria	Random allocation	Concealed allocation	Similar baseline	Blinding subjects	Blinding therapists	Blinding assessors	85% retention	Intention to treat	Between-group comparisons	Point and variability measures	Total score
Benzing et al. (2016)	Yes	Yes	No	Yes	No	No	No	Yes	Yes	Yes	Yes	7
Bulten et al. (2022)	Yes	Yes	Yes	Yes	No	No	No	Yes	Yes	Yes	Yes	8
Chou et al. (2020)	Yes	Yes	No	Yes	No	No	Yes	Yes	Yes	Yes	Yes	8
Crova et al. (2014)	Yes	Yes	No	Yes	No	No	No	Yes	Yes	Yes	Yes	7
Egger et al. (2018)	Yes	Yes	Yes	Yes	No	No	No	Yes	Yes	Yes	Yes	8
Egger et al. (2019)	Yes	Yes	No	Yes	No	Yes	No	Yes	Yes	Yes	Yes	8
Flynn and Richert (2017)	No	Yes	No	Yes	No	No	No	Yes	Yes	Yes	Yes	6
Gao et al. (2019)	Yes	Yes	Yes	Yes	No	No	No	Yes	Yes	Yes	Yes	8
Giordano and Alesi (2022)	Yes	Yes	No	Yes	No	No	No	Yes	Yes	Yes	Yes	7
Jäger et al. (2015)	Yes	Yes	Yes	Yes	No	No	No	No	Yes	Yes	Yes	7
Kolovelonis et al. (2023)	Yes	Yes	No	Yes	No	No	No	Yes	Yes	Yes	Yes	7
Layne et al. (2020)	Yes	Yes	No	Yes	No	No	No	Yes	Yes	Yes	Yes	7
Mavilidi et al. (2023)	Yes	Yes	No	Yes	No	No	Yes	Yes	Yes	Yes	Yes	8
Meijer et al. (2020)	Yes	Yes	Yes	Yes	No	No	No	Yes	Yes	Yes	Yes	8
Meijer et al. (2022)	Yes	Yes	Yes	Yes	No	No	No	Yes	Yes	Yes	Yes	8
Oppici et al. (2020)	Yes	Yes	Yes	Yes	No	No	Yes	Yes	Yes	Yes	Yes	9
Pesce et al. (2016)	Yes	Yes	No	Yes	No	No	No	Yes	Yes	Yes	Yes	7
Robinson et al. (2022)	Yes	Yes	No	Yes	No	No	Yes	No	Yes	Yes	Yes	7
Schmidt et al. (2015)	Yes	Yes	No	Yes	No	Yes	No	Yes	Yes	Yes	Yes	8
Schmidt et al. (2020)	Yes	Yes	No	Yes	No	Yes	No	Yes	Yes	Yes	Yes	8
van der Niet et al. (2016)	Yes	No	No	Yes	No	No	No	Yes	Yes	Yes	Yes	6
van der Fels et al. (2020)	Yes	Yes	Yes	Yes	No	No	No	Yes	Yes	Yes	Yes	8
Vazou and Mavilidi (2021)	Yes	Yes	Yes	Yes	No	No	Yes	Yes	Yes	Yes	Yes	9
Mean score												7.6

## 3 Results

**Figure 1:**
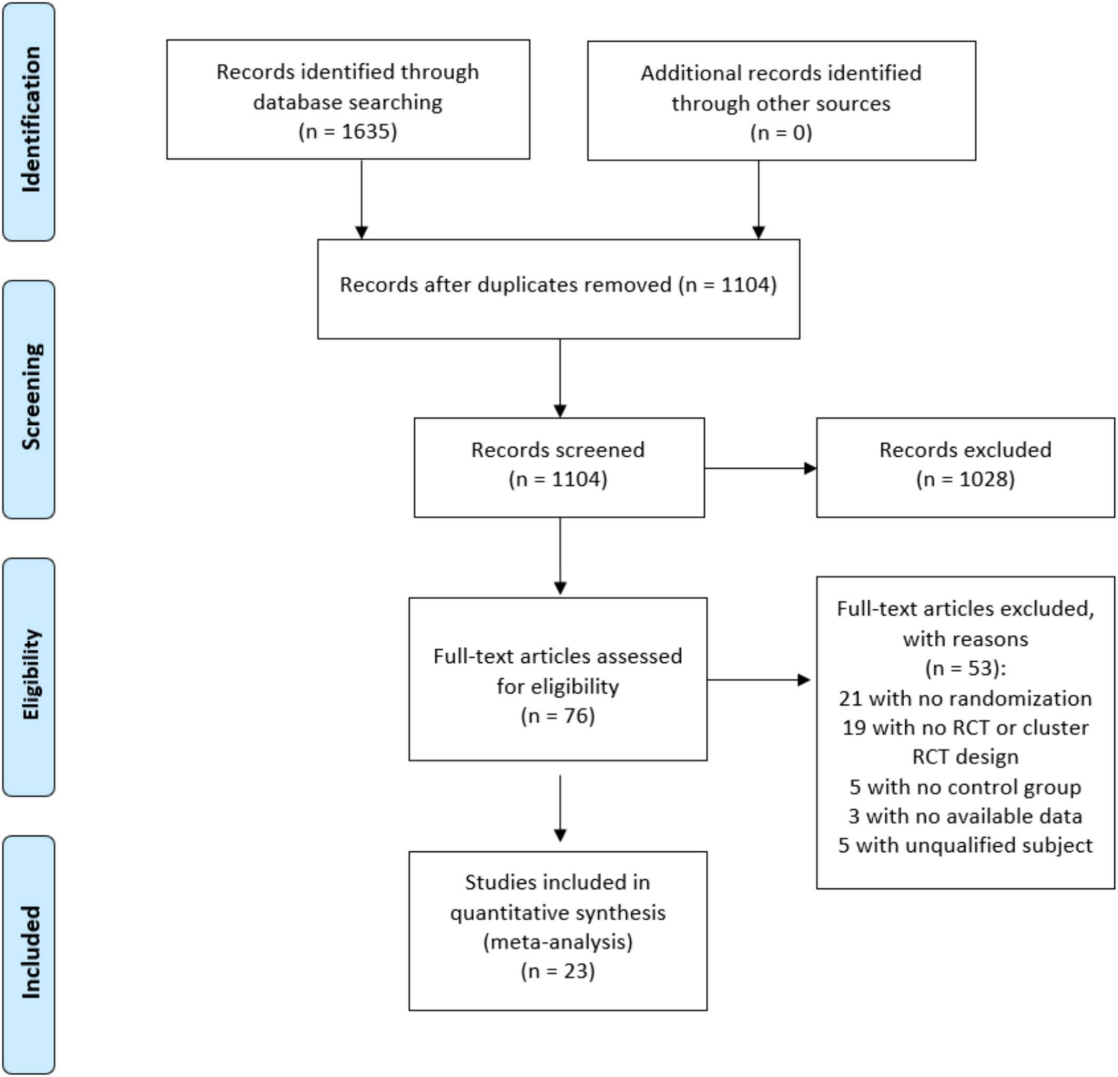
Flowchart of literature search and study selection.

The initial search retrieved 1,635 peer-reviewed articles. After removing duplications and reviewing the titles, 1,104 articles were eligible for further screening. Through a careful reading of the abstracts, 76 articles were eligible for thorough examination. Finally, 23 studies were included in the meta-analysis. Among the initial pool of 76 articles, 53 were excluded because they had no control group, lacked randomization, no RCT or cluster RCT design, lacked available data or had unqualified samples. Figure 1 displays the flow of study selection.

### 3.1 Study characteristics

Overall, 23 studies were included in the meta-analysis and the study characteristics are summarized in Table 1. Nine studies were RCTs and 14 studies were cluster RCTs. The total sample included 2,857 children and adolescents aged from 4 to 16 years. There were 23 studies investigating the effects of cognitively engaging PA on core EFs, of which, 13 studies examined all three core features, three examined 2 features, and 7 examined a single feature. Regarding the exercise intervention protocols, there were 22 studies with sessions over 20 min, 14 studies with more than two sessions per week and 19 studies with durations over 6 weeks. The major confounding factors considered in the studies included intervention modality, duration, frequency, and session length.

### 3.2 Effects of cognitive engagement PA on EFs

**Figure 2:**
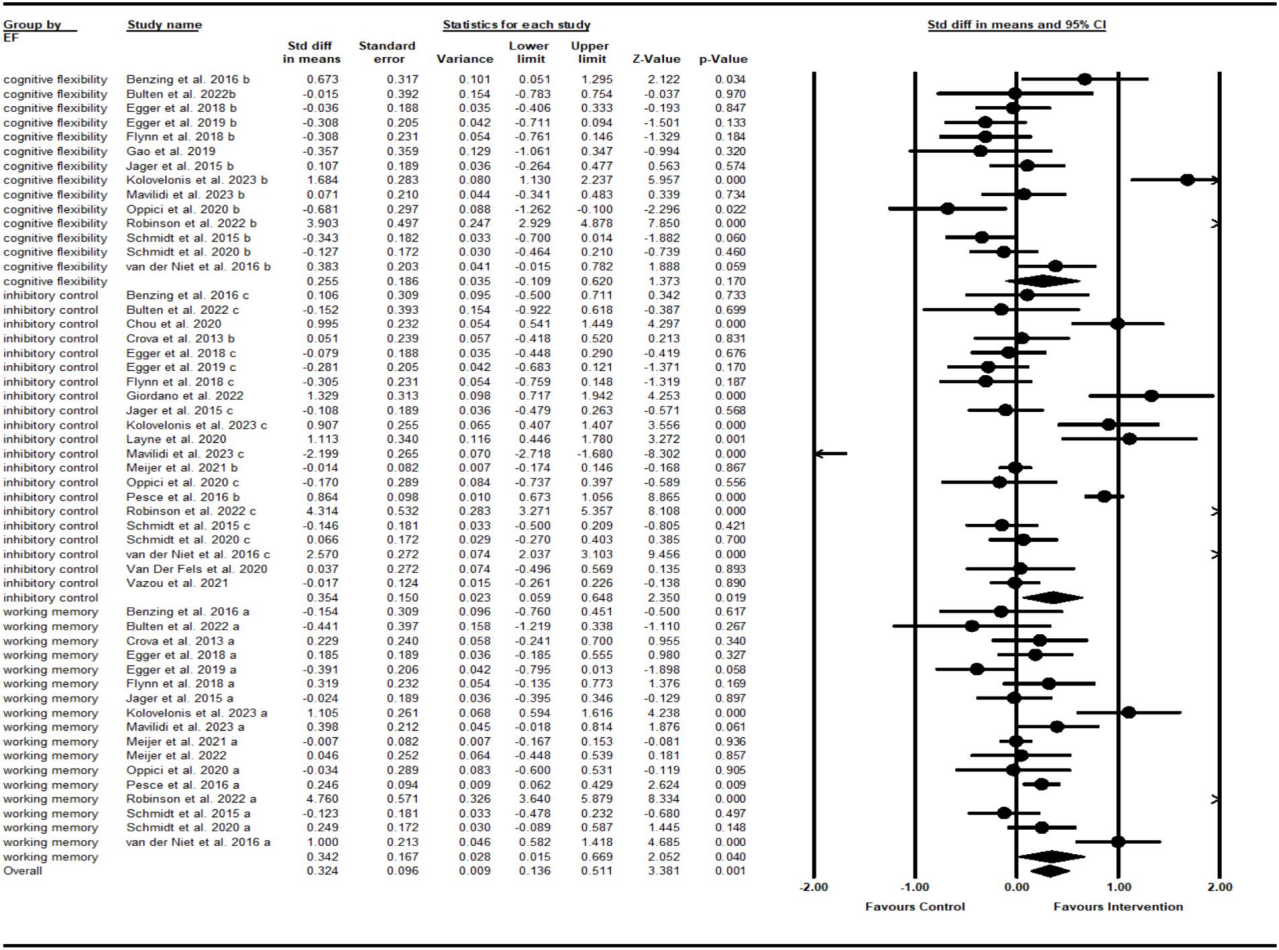
Forest plot for meta-analysis regarding the effect of cognitively engaging PA interventions on different EF domains.

As illustrated by Figure 2, the pooled SMD of over EF was 0.32 (95% CI 0.14 to 0.51, *p* = 0.001), with large heterogeneity (*I*^2^ = 91.1%, *p* < 0.001). Regarding core EFs, a total of 23 studies were included in the analysis. Thirteen of these studies evaluated three core features, three assessed 2 features, and 7 examined a single feature. The SMD was 0.26 (95% CI −0.11 to 0.62) for cognitive flexibility, with large heterogeneity (*I*^2^ = 89.1%, *p* < 0.001); 0.35 (95% CI 0.06 to 0.65) for inhibitory control, with large heterogeneity (*I*^2^ = 94.1%, *p* < 0.001); and 0.34 (95% CI 0.02 to 0.67) for working memory, with large heterogeneity (*I*^2^ = 86.1%, *p* < 0.001). The high heterogeneity for core EFs reflects the importance of taking various moderator factors into consideration when analyzing the effects of cognitively engaging PA.

### 3.3 Moderator analysis

Table 3 summarizes the results from the moderator analysis and subgroup analyses for potential factors on EFs. In the subgroup analysis, the effect of cognitively engaging PA on EFs was significantly moderated by intervention modality, frequency, duration, and session length. Firstly, a total of 52 studies evaluated the effects of different modalities of PA interventions on EFs. Among them, 24 studies investigated the intervention effects of FMS, 20 studies assessed the impact of PA games interventions, and 8 studies examined the effects of exergaming intervention modalities. The SMD for modality of FMS was 0.49 (95% CI 0.22 to 0.77) indicating large heterogeneity (*I*^2^ = 94.3%, *p* < 0.001), whereas the SMD for PA games was 0.20 (95% CI −0.10 to 0.49), with large heterogeneity (*I*^2^ = 86.1%, *p* < 0.001), and 0.13 (95% CI −0.36 to 0.61) for exergaming, with moderate heterogeneity (*I*^2^ = 67.3%, *p* = 0.003). With respect to the exercise duration, 19 studies assessed intervention periods longer than 6 weeks, while 33 studies examined intervention durations of 6 weeks or less. The SMD for duration >6 weeks was 0.38 (95% CI 0.15 to 0.62), with large heterogeneity (*I*^2^ = 91.0%, *p* < 0.001), whereas the SMD for ≤6 weeks was 0.22 (95% CI-0.08 to 0.52), with large heterogeneity (*I*^2^ = 91.4%, *p* < 0.001). With regard to the intervention frequency, 14 studies evaluated intervention frequencies of more than twice a week, while 38 studies examined exercise frequencies of 2 weeks or less. The SMD for frequency > 2 times/week was 0.89 (95% CI 0.53 to 1.26), with large heterogeneity (*I*^2^ = 94.4%, *p* < 0.001), and the SMD for ≤2 times/week was 0.12 (95% CI −0.09 to 0.34), with large heterogeneity (*I*^2^ = 89.1%, *p* < 0.001). With respect to the session length, 22 studies assessed session length longer than 20 min, while 30 studies examined session length of 20 min or less. The SMD for session length > 20 min was 0.37 (95% CI 0.10 to 0.65), with large heterogeneity (*I*^2^ = 91.1%, *p* < 0.001), whereas the SMD for ≤20 min was 0.28 (95% CI 0.04 to 0.52), with large heterogeneity (*I*^2^ = 91.0%, *p* < 0.001).

**Table 3:** Moderator analysis of cognitively engaging PA and EFs.

Categorical variables	Level	No. of studies	SMD	95%CI	*I*^2^%	Test of null (two-tailed)	
						*Z*-value	*p*-value
Modality	FMS	24	0.49	0.22 to 0.77	94.3	3.53	<0.001
	PA games	20	0.20	−0.10 to 0.49	86.1	1.32	0.188
	Exergaming	8	0.13	−0.36 to 0.61	67.3	0.51	0.607
Duration, week	>6	19	0.38	0.15 to 0.62	91.0	3.21	0.001
	≤6	33	0.22	−0.08 to 0.52	91.4	1.44	0.149
Frequency, times/week	>2	14	0.89	0.53 to 1.26	94.4	4.77	<0.001
	≤2	38	0.12	−0.09 to 0.34	89.1	1.13	0.260
Session length, min	>20	22	0.37	0.10 to 0.65	91.1	2.64	0.008
	≤20	30	0.28	0.04 to 0.52	91.0	2.27	0.023

PA, physical activity; EF, executive function; FMS, fundamental movement skills.

### 3.4 Methodological quality assessment

The quality of the included studies is presented in Table 2. In general, those studies were scored as fairly high quality, with a mean quality score of 7.6, because they were (cluster) RCTs. Moreover, over half of the studies (18 out of 23) were school-based intervention, including active class, curricular PA, and extracurricular PA.

### 3.5 Publication bias

**Figure 3:**
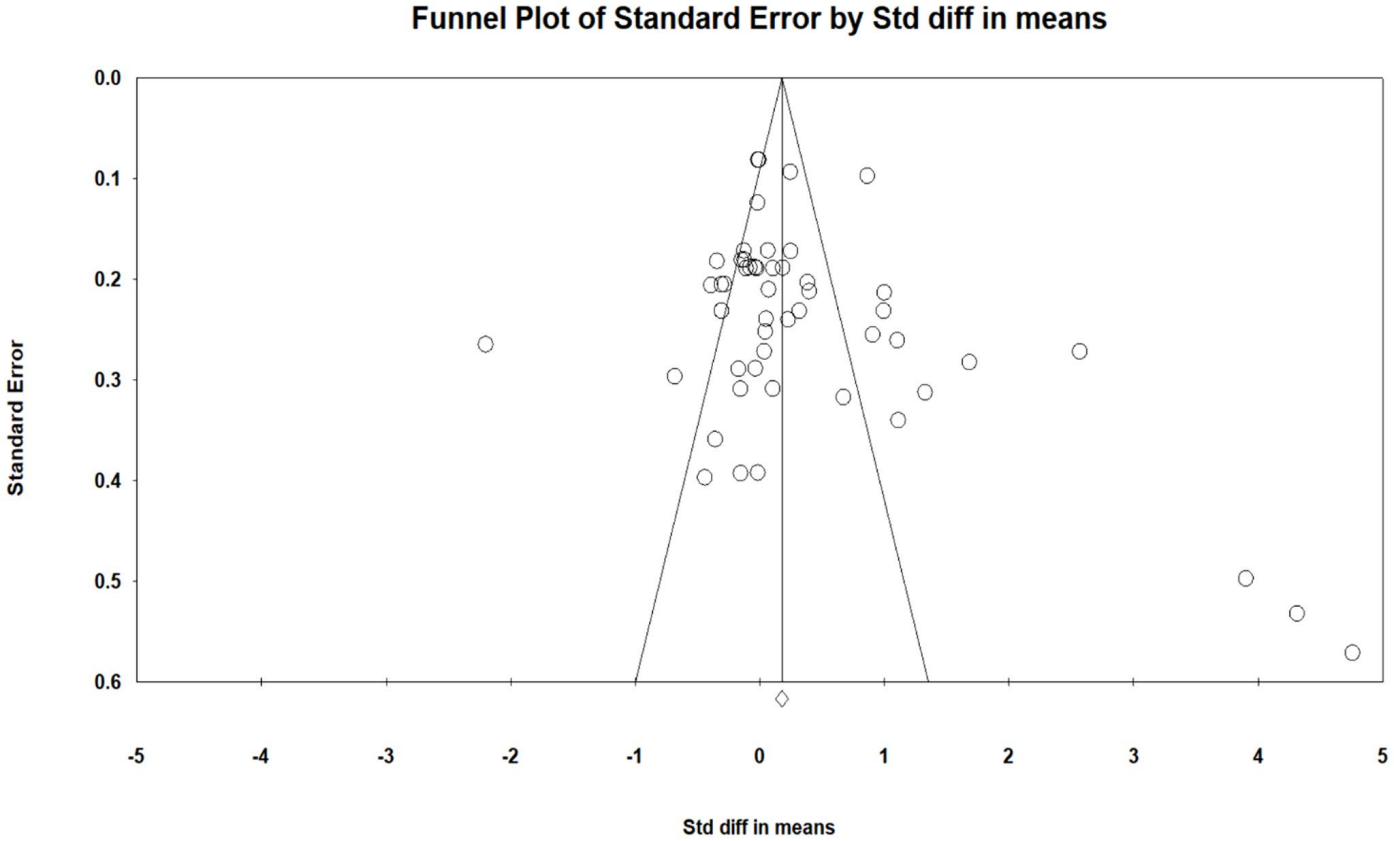
Funnel plot no publication bias cognitively engaging PA and EFs.

The funnel plot is presented in Figure 3. Egger’s test was used to assess publication bias (*t* = 1.67, df = 50, *p* = 0.10). The results indicated no evidence of publication bias for all included studies (Egger et al., 1997).

## 4 Discussion

### 4.1 Main study findings

The current review included 23 RCT studies which investigated the effects of cognitively challenging PA interventions on core EFs (cognitive flexibility, inhibitory control and working memory) in children and adolescents. Meta-analysis indicated a small but significant positive benefits of cognitive engagement PA on overall core EFs, particularly on inhibitory control. Moreover, our analysis identified several key moderating variables that influence the relationship between cognitive engagement PA and EFs, including intervention modality, duration, frequency, and session length.

The positive impact of cognitively engaging PA on overall EFs highlights the importance of integrating cognitive challenges within physical activities to enhance cognitive outcomes. The pooled SMD for overall EF improvement was 0.32, which, despite being modest, underscores a meaningful advancement in the cognitive capabilities of children and adolescents engaging in such interventions. Notably, the significant improvement in inhibitory control (SMD = 0.35) aligns with the theory that PA demanding cognitive engagement can strengthen brain regions responsible for self-regulation and attentional control (Best, 2010).

### 4.2 Analysis of moderating variables between cognitive engagement PA and EFs

The modality of PA intervention significantly influences its effectiveness on EFs. Various studies have shown that interventions combining physical and cognitive tasks, such as FMS and PA games, yield more significant improvements in EFs compared to traditional PA. Best (2010) highlighted that aerobic exercises integrated with cognitive challenges, like sports requiring strategic planning or movement routines involving memorization, show greater enhancements in EFs. This is corroborated by Ferreira Vorkapic et al. (2021), who emphasized that activities requiring real-time decision-making and problem-solving, such as cognitive games and complex sports drills, are particularly effective. For instance, exergaming, which involves active video games that require physical movement and cognitive processing, has been shown to improve inhibitory control and cognitive flexibility (Anzeneder et al., 2023). Furthermore, a review by Tomporowski et al. (2015) found that PA interventions with cognitive engagement had positive effects on EFs, especially in tasks demanding higher-order cognitive functions. This indicates that the inclusion of cognitive demands in PA is a critical factor in enhancing EFs in children and adolescents. Therefore, it is essential for PA interventions to incorporate complex cognitive elements to maximize their impact on EFs.

The duration and frequency of PA interventions are also crucial moderators of their impact on EFs. Long-term interventions tend to produce more substantial improvements in cognitive functions compared to short-term interventions. For example, a study by Hillman et al. (2014) demonstrated that a 9-month intervention significantly enhanced EFs, particularly in areas of working memory and cognitive flexibility. In contrast, shorter interventions, typically lasting less than 6 weeks, often show mixed results due to insufficient exposure (de Greeff et al., 2018). This is consistent with findings from Pesce et al. (2016), who reported that sustained cognitively engaging PA interventions over several months resulted in more pronounced cognitive benefits. The current meta-analysis supports these findings, indicating that interventions lasting more than 6 weeks generally have a more significant impact on cognitive flexibility and working memory. Additionally, the frequency of interventions plays a vital role; those conducted more than twice a week tend to yield better outcomes. Ludyga et al. (2018) suggested that regular engagement in cognitively demanding PA facilitates sustained cognitive engagement and neural adaptations. Frequent and prolonged exposure to such activities likely promotes neuroplasticity and cognitive development, which are essential for improving EFs (Kvalø et al., 2017). Therefore, to achieve significant cognitive benefits, PA interventions should be designed to last longer and be administered more frequently.

The length of each PA session is another important factor affecting its efficacy. Sessions longer than 20 min have been shown to be more beneficial for cognitive outcomes compared to shorter sessions. Wang et al. (2023) demonstrated that extended PA sessions provide sufficient time for both physical exertion and cognitive engagement, which are necessary for stimulating cognitive improvements. Longer sessions allow for more complex cognitive tasks and greater mental effort, leading to better EF outcomes (Davis et al., 2011; Kvalø et al., 2017). Additionally, longer sessions may enhance the cardiovascular and metabolic responses needed for cognitive benefits, as suggested by de Greeff et al. (2016). This is further supported by a study conducted by Gallotta et al. (2012), which found that longer PA sessions resulted in significant improvements in cognitive performance due to sustained cognitive engagement. The current meta-analysis corroborates these findings, showing that PA sessions exceeding 20 min are associated with significant improvements in inhibitory control and working memory. Therefore, to maximize cognitive benefits, it is crucial for PA sessions to be designed with sufficient length to ensure comprehensive cognitive and physical engagement.

### 4.3 Potential mechanisms of cognitive engagement PA and EF

Cognitive promotion associated with the cognitively engaging PA interventions implies potential changes in the central nervous system. Neuroimaging evidence has shown that cognitive activities during exercise stimulate blood flow and activation in prefrontal regions, thus resulting in better cognitive performance than simple aerobic exercises (Hillman et al., 2009; Liu et al., 2023). Cognitively challenging PA also increases the release of the brain-derived neurotrophic factor (BDNF) which plays an essential role in enhancing synaptic plasticity and improving cognitive performance (Voss et al., 2011; Winter et al., 2007). Schmidt et al. (2015) found that children who participated in cognitively engaging PA exhibited greater increases in BDNF and corresponding improvements in EF tasks compared to those involved in standard aerobic exercises.

In addition to the explanations at the neural level, researchers proposed potential mechanisms from psychological perspectives. Cognitively engaging PA interventions may enhance EF by improving self-regulation and motivational states. Engaging in activities that require constant cognitive input, such as strategy-based games or problem-solving tasks, helps children and adolescents practice self-control, attention regulation, and goal-directed behavior (Kolovelonis and Goudas, 2022). These activities often require participants to navigate complex rules, make quick decisions, and adjust strategies in real-time, which mirrors the demands of EF. Consequently, regular participation in such activities can translate into better EF by continuously challenging and refining these cognitive processes. Moreover, the integration of cognitive and physical elements in PA interventions can enhance motivation and enjoyment, which are critical factors for sustained engagement and practice. Studies have shown that when children find activities enjoyable and intrinsically rewarding, they are more likely to persist, thereby gaining more practice and potential benefits for EF (Kolovelonis et al., 2023; Tomporowski et al., 2008). This increased engagement not only promotes physical fitness but also provides repeated opportunities to exercise cognitive skills in a dynamic and interactive context, leading to cumulative benefits for EF.

### 4.4 Implications for educational practices and interventions

Given the findings from this meta-analysis, several practical implications emerge for integrating cognitively engaging PA into educational settings. Notably, recent studies underscore the importance of considering the intensity, duration, and frequency of PA to maximize its benefits. Research has consistently shown that moderate to vigorous intensity PA, particularly when sustained over longer periods, yields substantial improvements in EFs such as working memory, cognitive flexibility, and inhibitory control (Morales et al., 2024; Wen et al., 2018). For instance, a meta-analysis by Vazou et al. (2019) highlighted that aerobic exercises, motor skill training, and cognitively engaging activities all positively impact cognitive performance in young individuals.

Furthermore, the type of PA intervention is critical. High-Intensity Interval Training (HIIT), for example, has demonstrated considerable promise due to its combination of short bursts of intense exercise with brief recovery periods. This format not only enhances physical fitness but also challenges cognitive processes, thereby improving EFs (Gilson et al., 2023; Leahy et al., 2020). Similarly, cooperative and playful activities, which integrate social interaction and cognitive engagement, can significantly enhance motivation and enjoyment, leading to sustained participation and greater cognitive benefits (Wang et al., 2024; Wei et al., 2024). Such interventions are particularly effective in educational settings, where the dual goals of promoting physical health and cognitive development can be seamlessly integrated into the curriculum.

Educators and families can leverage these insights to design PA interventions that are both engaging and beneficial. For instance, incorporating game-based activities that require strategic thinking and problem-solving can make physical education classes more enjoyable and cognitively stimulating (Vita-Barrull et al., 2023). These activities could range from team sports that necessitate real-time decision-making to classroom exercises that combine physical movement with academic content. The key is to ensure that the PA is not merely about physical exertion but also includes elements that challenge students’ cognitive abilities, thereby promoting holistic development (Schmidt et al., 2015). The integration of cognitively engaging PA within the school day holds significant potential for enhancing academic performance and cognitive development. By focusing on the intensity, duration, frequency, and type of exercise, educators can design interventions that provide comprehensive benefits to students. This approach not only supports physical health but also fosters essential cognitive skills, preparing students for both academic success and lifelong well-being.

### 4.5 Strengths and limitations

One of the advantages of this meta-analysis is the stringent inclusion criteria, which focus exclusively on RCT while excluding observational or longitudinal studies, as RCTs are considered the gold standard for intervention studies. This rigorous approach ensures the validity of the study results, enhancing the reliability of causal inferences. Additionally, the use of SMD to quantify effect sizes allows for the comparison of outcomes across different measures, providing a more consistent and interpretable analysis. Another strength is the significant effect observed in the moderator analysis conducted in this study. By examining variables such as intervention modality, duration, frequency, and session length, this meta-analysis identifies key factors that influence the effectiveness of cognitively engaging PA interventions.

A limitation of this review is the high heterogeneity in the participants of the included studies. The research works involve preschool children (Gao et al., 2019; Giordano and Alesi, 2022; Mavilidi et al., 2023; Schmidt et al., 2020; Vazou and Mavilidi, 2021), school-aged children (Crova et al., 2014; Egger et al., 2019; Flynn and Richert, 2017; Kolovelonis et al., 2023), and adolescents (Benzing et al., 2016; Robinson et al., 2022; Schmidt et al., 2015). Cognitive development is age-and sex-specific, suggesting that the effect of physical exercise may be different by age and sex. Given that both factors were not considered in data synthesis and analysis, it is important to interpret the results with cautions.

## 5 Conclusion

This systematic review and meta-analysis reveal that cognitively engaging PA interventions have a small yet significant positive impact on EFs in children and adolescents, particularly on inhibitory control. The analysis identified key moderating variables, such as intervention modality, duration, frequency, and session length, which influence the effectiveness of these interventions. Despite substantial heterogeneity among the included studies, the findings suggest that longer and more frequent interventions yield greater cognitive benefits. Understanding the underlying mechanisms of these cognitive benefits remains crucial, warranting further explorations through neuroimaging and neurophysiological research. Overall, incorporating cognitive elements into PA programs shows promise for enhancing EFs, providing valuable insights for future educational and clinical applications. On the other hand, substantial variability in age and sex of the participants highlights the need for practitioners and researchers to interpret the results with cautions.

## Data Availability

The original contributions presented in the study are included in the article/supplementary material, further inquiries can be directed to the corresponding author.
